# Monitoring Soil and Ambient Parameters in the IoT Precision Agriculture Scenario: An Original Modeling Approach Dedicated to Low-Cost Soil Water Content Sensors

**DOI:** 10.3390/s21155110

**Published:** 2021-07-28

**Authors:** Pisana Placidi, Renato Morbidelli, Diego Fortunati, Nicola Papini, Francesco Gobbi, Andrea Scorzoni

**Affiliations:** 1 Dipartimento di Ingegneria, University of Perugia, 06125 Perugia, Italy; diego.fortunati@studenti.unipg.it (D.F.); nicola.papini@studenti.unipg.it (N.P.); francesco.gobbi@studenti.unipg.it (F.G.); andrea.scorzoni@unipg.it (A.S.); 2 Dipartimento di Ingegneria Civile e Ambientale, University of Perugia, 06125 Perugia, Italy; renato.morbidelli@unipg.it

**Keywords:** soil water content, sensor networks, distributed sensing, IoT measurements, Precision Agriculture, moisture sensor, wireless communication, LoRa, LoRaWAN™

## Abstract

A low power wireless sensor network based on LoRaWAN protocol was designed with a focus on the IoT low-cost Precision Agriculture applications, such as greenhouse sensing and actuation. All subsystems used in this research are designed by using commercial components and free or open-source software libraries. The whole system was implemented to demonstrate the feasibility of a modular system built with cheap off-the-shelf components, including sensors. The experimental outputs were collected and stored in a database managed by a virtual machine running in a cloud service. The collected data can be visualized in real time by the user with a graphical interface. The reliability of the whole system was proven during a continued experiment with two natural soils, Loamy Sand and Silty Loam. Regarding soil parameters, the system performance has been compared with that of a reference sensor from Sentek. Measurements highlighted a good agreement for the temperature within the supposed accuracy of the adopted sensors and a non-constant sensitivity for the low-cost volumetric water contents (VWC) sensor. Finally, for the low-cost VWC sensor we implemented a novel procedure to optimize the parameters of the non-linear fitting equation correlating its analog voltage output with the reference VWC.

## 1. Introduction

In recent years, the rapid development and broad application of the IoT (Internet of Things) concept pushed towards the improvement of best practices in Wireless Sensor Networks (WSNs) [1] in Precision Agriculture (PA) applications, also relevant to Greenhouses [2,3]. Smart, cheap, and powerful connected sensor nodes (things) are transforming from stand-alone devices to parts of collaborative systems [4,5]. Data are stored, aggregated, and analyzed to improve the precision of temporal-spatial parameters on croplands [6,7]. WSN could be made of simple and cheap components: the results provided by complex technology systems are not necessarily significantly better than the results derived from a combination of descriptive statistics and simple sensors: intrinsic limitations of the sensing element could be overcome [8] also providing the measurement readout in a digital format [9].

Currently, the sensor networks that characterize the IoT technology have the main purpose of collecting data from the surrounding world on intelligent systems for environmental applications [10,11]. Additionally, in cloud computing approaches, the collected data are analyzed, processed, and used to undertake the correct decisions to optimize natural resources: it follows that the set of sensors, devices, and storage systems, by which the IoT is composed, is very similar to a huge, distributed measurement system, as clearly outlined in [12]. The management of such complex systems is part of the present Big Data paradigm. Details on sampling techniques, distributed smart monitoring, and mathematical theories of distributed sensor networks can be found in [1,13].

In [14] the authors made a very good literature review on the use of machine learning (ML), a subset of artificial intelligence having a considerable potential to handle numerous challenges in the establishment of knowledge-based farming systems. In the paper, the authors considered four main generic categories of applications: crop, water, soil, and livestock management. In the paper the authors underlined also that (i) the majority of the journal papers focused on crop management [15,16]; (ii) several ML algorithms have been developed to handle the heterogeneous data coming from agricultural fields [17]; (iii) multispectral or RGB images constituted the most common input for ML algorithms, thus justifying the broad usage of Convolutional Neural Networks due to their ability to handle this type of data more efficiently [18,19]. Moreover, a wide range of parameters regarding the weather as well as the soil, water, and crop quality was used. The most common means of acquiring measurements for ML applications was remote sensing, including imaging from satellites, unmanned vehicles (both ground (UGV) and aerial (UAV)), while in situ and laboratory measurements were also used [20].

Very good reviews of the most common sensors used in agriculture applications are reported in [21,22]. In [23], agricultural sensors have been divided into three main classes: physical property type sensors, biosensors, and micro-electro-mechanical system (MEMS) sensors. Near and remote sensing techniques use IoT sensors for monitoring multiple parameters, such as soil water content, temperature, and pH level, air humidity, temperature, light, and pressure [23,24,25,26]. The determination of soil water content is a subject of great value in different scientific fields, such as agronomy, soil physics, geology, soil mechanics, and hydraulics. Physical, mineralogical, chemical, and biological properties are also involved. Moreover, soil water content measurements could be affected by soil temperature [27]. Ambient Relative Humidity (RH) affects leaf growth, photosynthesis, pollination rate, and finally crop yield. A prolonged dry environment or high temperature can make the delicate sepals dry quickly and cause the death of flowers before maturity. Hence it is very crucial to control air humidity and temperature. Recent technological advances have enabled real-time sensors to be used directly in the soil, wirelessly transmitting data without the need for human intervention. It is now possible to set up a large number of low-cost devices not only capable of transducing a physical quantity of interest but also of performing some post-processing on raw data to extract useful information, fully complying with current regulations [27,28,29]. Due to the rapid advancement of technologies, the size and the cost of sensors have been reduced, making WSN the foremost driver of PA [30].

While most previously cited parameters (including soil temperature) can reliably be monitored through low-cost sensors available in the market, the experimental and accurate determination of soil water content with low-cost sensors is still an issue. A summary of state of the art on soil water content measurement techniques has been reported in [31]. The prices of the most reliable soil water content sensors range between USD 150 and USD 5000, thus positioning these sensors far from the IoT world. Instead, the reliability of very low-cost soil water content sensors easily purchasable in the worldwide internet market is still a matter of scientific debate [8,32,33,34,35,36,37] as further highlighted in the next sections.

In this scenario, the objectives of the present work can be summarized as follows:

- Acquisition of basic physical parameters of plants and ambient with low-cost sensors: soil water content and temperature, greenhouse ambient RH, temperature, and light. Even if the present paper will mostly be focused on soil water content and most parameters will not be discussed, the availability of multiple parameters could be exploited in the future to build a more intelligent system by using machine learning algorithms.
- Availability of a modular system built with cheap off-the-shelf components also providing capabilities for automation and management of plant irrigation.
- Comparison of the performance of a very low-cost soil moisture sensor with a commercially available expensive system using two different types of soil with an original modeling approach which helps us to compare measurement results taken at different soil depths.

## 2. IoT Architecture in Precision Agriculture Scenario

### 2.1. Water Waste and Agriculture

The integration of information and control technologies in agriculture processes is known as Precision Agriculture. To obtain the greatest optimization and profitability PA adapts common farming techniques to the specific conditions of each point of the crop, by applying different technologies: micro-electro-mechanical Systems, Wireless Sensor Networks (WSN), computer systems, and enhanced machinery. PA optimizes production efficiency, increases quality, minimizes environmental impact, and reduces the use of resources (energy, water) [38].

The application of IoT allows farmers to boost the production process through plantation monitoring, soil and water management, irrigation scheduling, fertilizer optimization, pest control through chemicals as herbicides, delivery tracking. These tasks can be accomplished by using data from sensors, images, agricultural information management systems, global positioning systems (GPS), and communication networks. This integration results in the optimization of scarce resources [39].

Atmospheric changes and, in particular, the sudden rise in temperatures worsen the problem of searching for fresh water and water storage resources [40]. These problems are exacerbated in countries characterized by drought and rare rainfall, where the difficulty in finding the raw material prevents the development of crops (e.g., the California drought [41]). The scarcity of water in some regions of the world has led farmers to re-evaluate conventional agricultural methods to reduce waste. To this purpose, innovative systems and methods aimed at PA are needed, where sensor technology, electronic and communication engineering, and farming machinery are blended with cloud storage and computing. If on one hand, there is a tendency to optimize traditional irrigation systems using intelligent drip systems [42,43,44], on the other hand, systems and sensors [8] are sought to measure the soil water content in real-time [45]. In this way, it is possible to know the exact time and the specific position of soil that requires irrigation. However, regardless of all the advances in the IoT domain, the adoption of PA has been limited to some developed countries. Because of the lack of resources, remote sensing-based techniques to monitor crop health are uncommon in developing countries, thus resulting in a loss of yield. [25]

The development of WSN applications in PA makes it possible to increase efficiency, productivity, and profitability in many agricultural production systems while minimizing unintended impacts on wildlife and the environment. The real-time information obtained from the fields can provide a solid base for farmers to adjust strategies at any time. Instead of making decisions based on some hypothetical average conditions, which may not exist anywhere, a precision farming approach recognizes differences and adjusts management actions accordingly [46].

The combination of WSN, which are cheaper to implement than wired networks [29], with intelligent embedded systems and applying on this combination the technology of ubiquitous systems [40], leads to the development of the design and implementation of low-cost systems for monitoring agricultural environments, suitable for developing countries and difficult access areas.

### 2.2. IoT Architectures

Wireless Sensor Networks (WSNs) have extensively been adopted in agriculture [47] as well as in livestock farming [48] due to installation flexibility especially when wireless transmission introduces a significant reduction and simplification in wiring and harness [30,49]. In addition, greenhouse technology profits from this technology through automation and informatization. In [46] an intelligent system, controlling and monitoring greenhouse temperature has been described, aiming at reducing consumed energy while maintaining good conditions that improve productivity. A review of the common wireless nodes and sensors capturing environmental parameters related to crops in the agriculture domain is reported in [29]. Agriculture in the Internet era is quickly becoming a data-intensive industry. Farmers need to gather and evaluate a massive amount of information from meteorological and physical sensors to increase production efficiency [50]. In [51] a description of a modular IoT architecture for several applications including but not limited to healthcare, health monitoring, and PA is reported. All the proposed subsystem choices used in that research are cheap off-the-shelf components with open-source software libraries.

### 2.3. Radio and Wireless Protocols in PA

The goal of optimizing water use for crops leads also to the development of automated irrigation systems. In [52] wireless sensors are linked by ZigBee radio transceivers, implementing a WSN where soil water content and temperature data are transferred. The wireless information unit also features a GPRS module that connects to a web server via the public mobile network. An online graphical application through Internet access devices allows operators to remotely monitor the information data. The feasibility of the implemented automated irrigation system was demonstrated. However, the total cost was high for some applications. The cost of each wireless sensor unit was ∼USD 100, whereas the wireless information unit cost was ∼USD 1800.

In addition to ZigBee, the IoT world is pushing new technologies. The Long-Range (LoRa) technology, originally developed for IoT, is investigated in [10] to demonstrate its use for implementing Distributed Measurement Systems. The LoRa wireless technology is designed for sending small packets at a low data rate (0.3–5.5 kbps) at relatively long distances. The protocol can be used in IoT nodes where energy efficiency is considered the most critical parameter.

The LoRaWAN™ protocol exploits the unlicensed radio spectrum in the Industrial, Scientific, and Medical band. Operating frequencies (433 MHz, 868 MHz, or 915 MHz) depend on the particular geographical region. Formally, LoRaWAN™ is a member of the low power LPWAN family, i.e., WAN wireless communications that are designed to minimize the power consumption while covering large areas but offering a relatively small bit rate. The specification defines the device-to-infrastructure of LoRa physical layer parameters and the LoRaWAN™ protocol. The LoRa physical layer or PHY exploits a Chirp Spread Spectrum (CSS) modulation. Fundamental keywords are low power transmission, low throughput, and optimum coverage. The LoRaWAN™ network architecture is deployed in a star-of-stars topology in which gateways relay messages between end-devices and a central network server. The gateways are connected to the network server via standard IP connections and act as a transparent bridge, simply converting RF packets to IP packets and vice versa [53].

LoRa is having success, as confirmed by the high number of papers adopting it (see e.g., [54,55,56,57,58] and references therein). The LoRa alliance sponsors the integration of LoRaWAN™ into the IoT, and some open implementations of network servers are available helping the constant growth of the LoRaWAN™ ecosystem. A fairly complete analysis of the scalability of networks based on LoRaWAN™ is reported in [58]. Employing analytic and simulation-based approaches, the authors explore the dimensions of the LoRa network configuration. The chosen spreading factor, a parameter directly related to the bitrate of the LoRa message, significantly depends on the number of sensors deployed in the field and on the transmission rate, given in packets/day.

## 3. System Architecture

**Figure 1:**
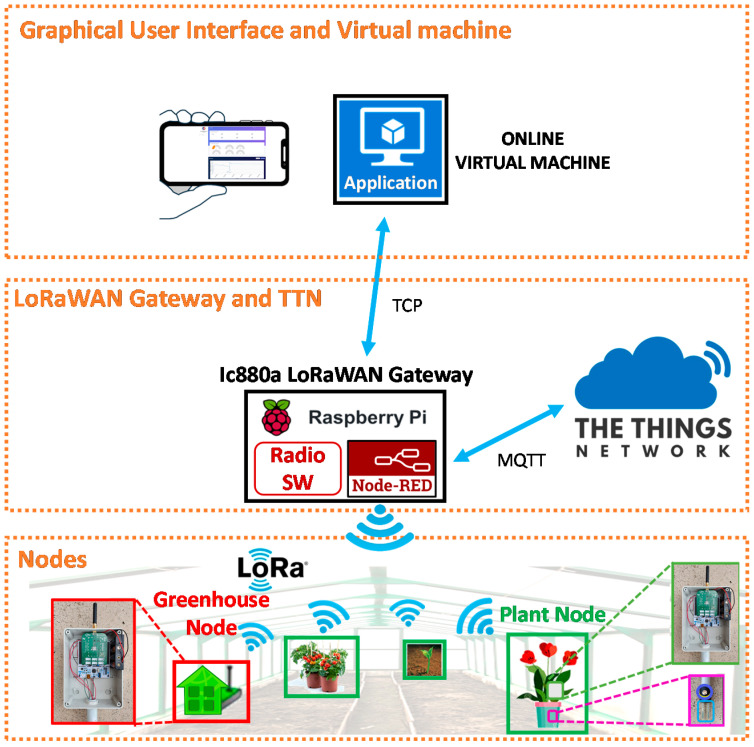
System architecture.

The modular design of the proposed approach splits the architecture into different layers (Figure 1): (*i*) wireless nodes (encompassing sensors, actuators, low-power embedded processor, battery), (*ii*) internet gateway/concentrator, and The Things Network (TTN) [59], a worldwide open-access LoRaWAN™ network, (*iii*) uplink and downlink connection, database applications, and user interface placed in a virtual machine in the cloud. Our layer structure is a simplified version for what is reported in [23] where our layer “*i*” corresponds to the perception layer, layer “*ii*” merges the *network* and the middleware layers, while our layer “*iii*” combines the common platform and the application layers. Details on the different blocks of Figure 1 will be given in the following sections.

### 3.1. Nodes

Two basic wireless nodes have been envisaged, each one equipped with Semtech SX1272 LoRa Radio: “Greenhouse Node” and “Plant Node”. A single “Greenhouse Node” is needed for a greenhouse. Instead, every plant to be monitored will feature a “Plant Node”.

**Figure 2:**
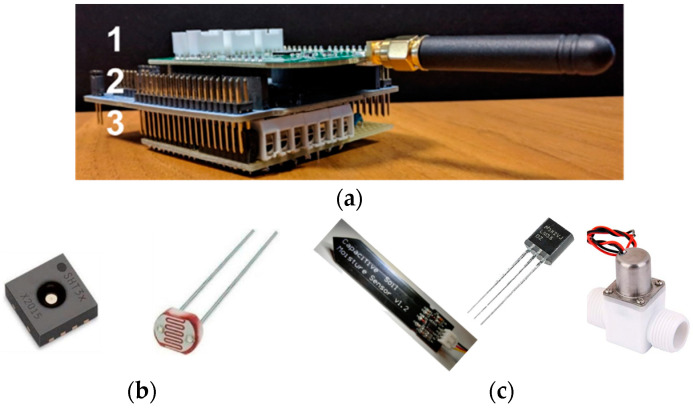
(**a**) Plant and Greenhouse node electronics and their actuator and sensors. (1) Semtech SX1272 LoRa Radio, (2) L152RE low power microcontroller, (3) Sensor/Actuator Interface Shield; (**b**) from left to right for the Greenhouse Node: Si7021 and Photoresistor. (**c**) from left to right for the Plant Node: “Capacitive Soil Moisture Sensor v1.2”, TMP36 or LM35 temperature sensor, and bistable solenoid valve.

Both node types share the same structure (Figure 2a), i.e., (1) low power, ARM-based STM32L152RE microcontroller hosted in a NUCLEO_L152RE board, (2) chirp spread spectrum SX1272 LoRa Radio, and (3) sensor shield.

The sensor shield of the Greenhouse Node (Figure 2b) provides the connections to (1) a Si7021, a common and widely used RH and temperature sensor, (2) a Photoresistor for light detection, and (3) a 4.8 V battery.

The Plant Node is dedicated to measuring the fundamental parameters of the soil, i.e., soil water content and soil temperature, and to soil watering. Its dedicated shield (Figure 2c) hosts a BD6212HFP H-bridge used for driving a bistable solenoid valve and a power feed interconnection to turn the sensors on and off. Moreover, it is connected to (1) a TMP36 or an LM35 temperature sensor, (2) a “Capacitive Soil Moisture Sensor v1.2” for measuring water content, (3) a bistable solenoid valve, and (4) a 4.8 V battery. Finally, it includes a 1 MΩ shunt resistor useful to correct a fabrication defect of the batch of sensor we received.

It is worth giving some details here on the design choices of this Plant Node. The use of an H-bridge and a bistable valve greatly helps in minimizing power consumption, as the valve will drain current only during switching. Power supply for temperature and water content sensors was delivered through the GPIOs of the microcontroller to feed the sensors only when a measurement must be accomplished. In fact, the maximum allowed current delivered by the GPIOs of the STM32L152RE microcontroller is more than enough for the low power sensors we adopted. However, it must be pointed out the present version of the system is designed to provide maximum flexibility and is not conceived for power optimization. For example, the STM NUCLEO boards still include the ST-LINK/V2-1 programming and debugging tool, whose power consumption is way larger than that of the main STM32L152RE microcontroller.

**Figure 3:**
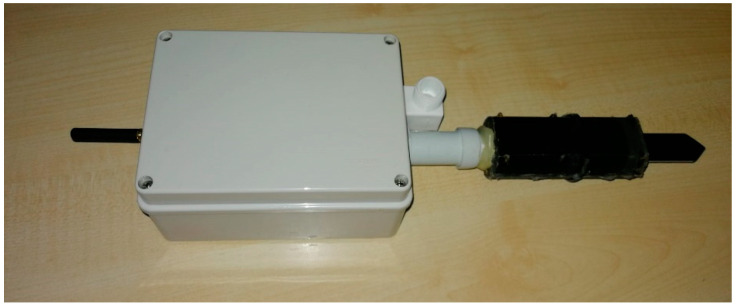
The final version of the Plant Node.

Several plant nodes have been manufactured (Figure 3). After some design steps, the present version of the Plant Node is composed of (*i*) a waterproof junction box (including all electrical and electronic components) connected with (*ii*) a 3D printed PET-G shell which protects the soil sensor and the temperature sensor.

Regarding the soil water content sensor, most papers presented in the literature either measure the capacitance of the soil, which, of course, depends on the water content, or adopt high frequency (around 100 MHz) AC measurements to characterize the dielectric constant of the soil [58]. Other papers describing low-cost IoT nodes adopt fork-like metallic sensors that, when used with a DC bias, mainly characterize the ionic content of the soil by measuring the electrical resistance between the two arms of the fork. AC could also be used with fork-like sensors to limit electrolysis and consequent metal electrode etching/degradation and DC ion currents in the soil. Examples can be found in GardenBot literature [60] or [27] and references therein.

For this prototype of the system, we use the commercial, blade-shaped, “Capacitive Soil Moisture Sensor v1.2” (also dubbed SKU: SEN0193 in its version 1.0 by DFROBOT [61]) for sensing water content in the soil. This sensor is undoubtedly the least expensive water content sensor in the market and was also exploited in other scientific papers (see for example [32,33]) and well-documented internet projects [62]. In [32] the authors found that this sensor did not perform acceptably in predicting soil moisture content in a laboratory soil mixture prepared by mixing organic-rich soil and vermiculite, while it can estimate soil water in gardening soil in the so-called “field capacity” range. In [33] the author linearly correlates the voltage provided by the sensor reading to the gravimetric moisture approximations, providing an effective relationship between the reading from the capacitive sensor and the water content in the soil. This calibration procedure demonstrated that low-cost capacitive-type soil moisture sensors are capable of predicting the water content in soils to a high degree of accuracy, with little required outside of the device itself, which is in direct contrast to the time it takes to traditionally measure the water content in soils.

**Figure 4:**
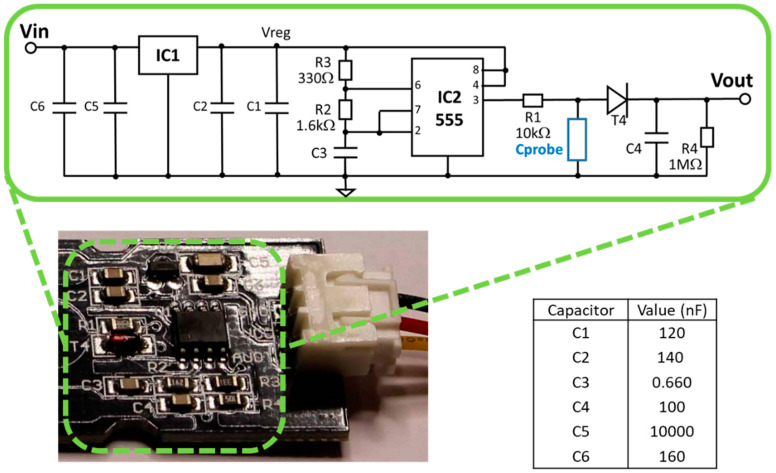
A detailed view of the printed circuit board component section with the electrical schematic of the “Capacitive Soil Moisture Sensor v1.2”. The reported resistance values are taken from the component labels while the capacitance values were measured using an HP4275A LCR meter. Cprobe is the variable capacitance of the coplanar capacitor printed on the circuit board. Due to a missing grounding line of the printed circuit board [8], in our measurements, a 1 MΩ shunt resistor has been directly connected to the Sensor/Actuator Interface Shield.

Being that the water content sensor is the hearth of our plant nodes and since a detailed data sheet is not available for the sensors, an accurate study of the sensor electronics was initially accomplished to get acquainted with the operation of the sensors [8]. A low dropout 3.3 V voltage regulator (omitted in a very recent version–v1.2 and v2.0–of this sensor) feeds a TL555I CMOS timer (Figure 4) which generates a trapezoidal waveform in astable mode running at about 1.5 MHz. The trapezoidal shape is because the operating frequency of the timer is pushed beyond the physical limit for the TL555I device, specified in the datasheet as guaranteed for 1.2 MHz in astable mode. On the other hand, the non-steep rising and falling edges of the waveform help in minimizing the electromagnetic interference possibly generated by the sensor and would be beneficial in the case of “CE” or “FCC” compliance certification.

The trapezoidal waveforms of nine sensors (S1, S2, S5, S6, S7, S9, S10, S13, and S14) were initially characterized to assess their uniformity. We discarded sensor S1 since its measured frequency *f* and duty cycle DC (1.22 MHz and 37.12% respectively) were very far from the average operating frequency and duty cycle of the other eight sensors (1.53 MHz and 34.48%, with sample standard deviations of 1% and 2.2%, respectively) [8], as reported in Table 1.

After this initial screening of the available samples of Capacitive Soil Moisture Sensor v1.2, we are confident that the measurement results of a single sensor chosen among other homogeneous samples represent the expected behavior of the whole family “S2, S5, S6, S7, S9, S10, S13, and S14”.

The TL555I timer supplies a passive circuit shown in Figure 4, composed of a first stage where the coplanar capacitor of the sensor Cprobe is low-pass connected with a 10 kΩ resistor. Then a peak detector provides the analog output signal that we acquire through the ADC of the microcontroller. Regarding sensor settling time, in [33] it was asserted this sensor should settle in 1–5 min, depending on the saturation level of the soil and how well the wet soil was mixed. We accomplished measurements with the Capacitive Soil Moisture Sensor v1.2 immersed in tap water and found that the output voltage could take up to one hour to reach the regime value. This could be due to a non-complete waterproofing of the sensor materials that likely incorporate water molecules. Therefore, the behavior of the Capacitive Soil Moisture Sensor v1.2 after initial watering could not be completely reproducible.

We underline that other more documented and reliable but also more expensive blade-shaped moisture sensors have been commercialized. Examples are the dielectric capacitance sensors ECH2O probe (Decagon Devices, Inc. Pullman, WA USA, now discontinued [63,64]) and the PROBE sensor [65], then modified to SMT100 ring-oscillator sensor (Truebner GmbH, Neustadt, Germany [66]) operating at approximately 150 MHz in water and 340 MHz in air.

A worst-case estimation of the overall cost of our plant node is roughly USD 60, where the most impacting figures are the microcontroller board and the LoRa shield.

#### 3.1.1. Soil Volumetric Water Content Fitting Equations

Water content measurements were previously accomplished in silica sandy soil with the Capacitive Soil Moisture Sensor v1.2 in conditions such that the dry unit weight $\gamma_{dry}=W_{s}/V$ ($W_{s}=$ dry soil weight, *V* = total volume of the soil) could be assumed as a constant [8]. It was demonstrated that this condition guarantees a monotonically decreasing *V*_s_ output voltage as a function of gravimetric water content (GWC), which was approximated using a 2nd order polynomial or an exponential function. In this paper, we will deal with volumetric water content (VWC) instead of GWC. However, the two parameters are proportional to each other for a given soil where the dry unit weight is constant. In the remainder of this paper, we will use the following exponential fitting equation between the output voltage *V_s_* of the Capacitive Soil Moisture Sensor v1.2 and the VWC:

(1) $$V_{s}=A\exp\left(-\frac{\mathrm{VWC}}{B}\right)+C,$$

(2) $$\mathrm{VWC}=B\;\ln\left(\frac{A}{V_{s}-C}\right)$$

being *A*, *B*, and *C* suitable constants. Other fitting equations were also adopted in the literature for the same sensor. In [32] a 3rd order polynomial function VWC = *f*(*V_s_*) was implemented. In [33] the following equation was used:

(3) $$\mathrm{VWC}=\frac{P}{V_{s}}-Q$$

being *P* = 2.48 V and *Q* = 0.72 for a soil composed of dried coconut coir. In the remainder of this paper, we will mainly deal with fitting Equations (2) and (3).

#### 3.1.2. Embedded Software Implementation of Nodes

**Figure 5:**
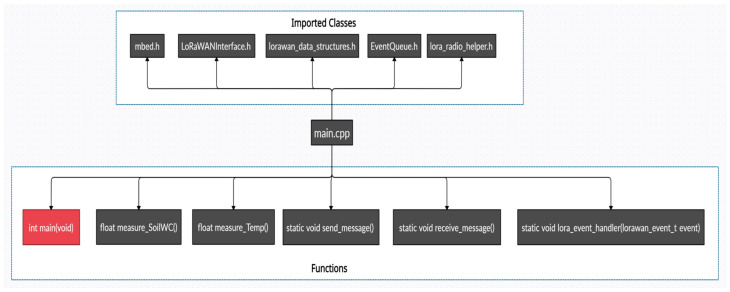
Embedded software implementation for the Plant Node.

The C++ code exploits ARM Mbed OS libraries. Mbed OS is an open-source Real-Time Operating System (RTOS) for the creation and deployment of IoT devices based on ARM processors. The code structure is outlined in Figure 5 for the case of the Plant Node.

The heart of the firmware is the *main.cpp* file. The header of main.cpp includes Mbed libraries (e.g., **EventQueue.h**) and LoRaWAN™ libraries. In particular, **LoRaWANInterface.h** encompasses the prototypes of the member functions managing the upper level of the LoRaWAN™ protocol stack, **lorawan_data_structures.h** includes LoRaWAN parameters, e.g., network and application key, datarate, duty cycle, antenna gain, buffer size, and SNR while **lora_radio_helper.h** regards the physical layer and selects the type of shield adopted in our system. The functions of **main.cpp** dedicated to the Plant Node are listed in the lower part of Figure 5. Among them, we cite the **lora_event_handler()** which manages the state machine of the LoRa events, the measuring functions for water content (**measure_SoilWC()**) and temperature (**measure_Temp()**), and the LoRa send and **send_message()** and **receive_message()** functions. The receive function also handles the bistable irrigation solenoid valve. In the case of the Greenhouse Node, the actual measuring functions regard ambient RH, temperature, and the ambient luminous flux.

**Figure 6:**
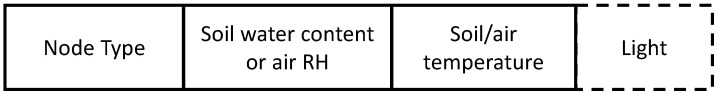
Packet structure.

Every node transmits a packet conforming to the structure defined in Figure 6. Depending on the node type (plant or greenhouse node), it will include different values. For example, the Plant Node features node type = 1 and transmits soil water content and temperature, while the greenhouse node is characterized by node type = 0 and transmits ambient RH, temperature, and light intensity.

### 3.2. The Things Network and Connection to the LoRaWAN™ Gateway

Routing and processing procedures of the LoRaWAN™ network are managed by The Things Network (TTN), acting as an active crossroad between the gateway and the application. For our application, we extensively use the TTN, a network server whose aim is building a global, worldwide open LoRaWAN™ network. They provide a set of open tools and a global, open network to build an IoT application at low cost, featuring maximum security and ready to scale. A secure and collaborative Internet of Things network is built through robust end-to-end encryption, spanning many countries around the globe. A network server does the complicated part in creating a LoRaWAN™ network (handling duplicate packets from multiple gateways, shunting data to servers, handling joins, etc.).

As shown in Figure 1, in the network architecture The Things Network is located between the LoRa concentrator/gateway and the applications. TTN is composed of three main structures: Router, Brokers, and Handler. The Router is in charge of managing the gateway’s status and of planning transmissions. Each Router is associated with one or more Brokers. The assignment of Brokers is to map a device to an application, to forward uplink messages to the proper application, and to forward downlink messages to the correct Router-Gateway path. A Handler is responsible for treating the data of different Applications. To do so, it deals with a Broker where it registers devices and applications. The Handler is also in charge of encrypting and decrypting data.

In our system, the Uplink connection to TTN is carried out by the Radio SW of the gateway (Figure 1) that publishes the node sensor data on a specific uplink topic of the TTN MQTT broker using an internet connection.

Then, through the well-known flow-based programming tool Node-RED [67] running on the Gateway, a specific device is allowed to communicate with the database installed in the virtual machine, as sketched in Figure 1.

**Figure 7:**
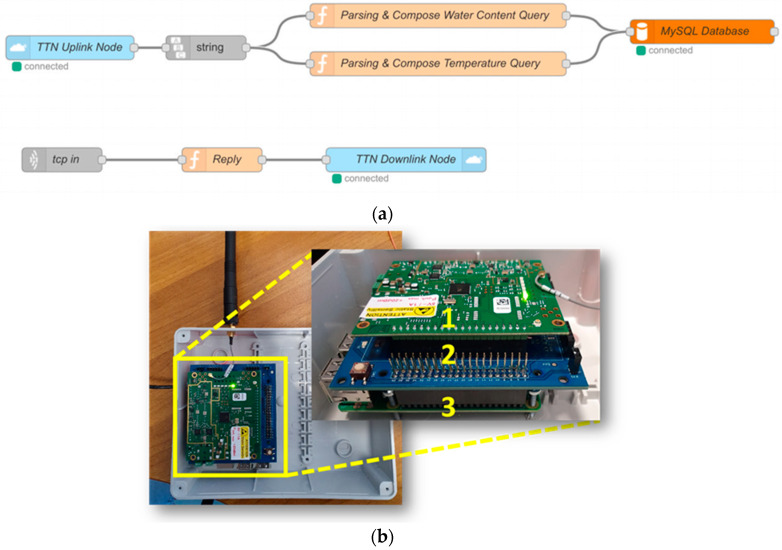
(**a**) The Node-RED flow running in the Raspberry of the Gateway. (**b**) The 8-channel iC880a /Concentrator, with interconnection backplane and Raspberry PI3, is mounted in a plastic box.

The Node-RED flow is composed of two sub-flows, an uplink, and a downlink flow, respectively (Figure 7a).

The uplink sub-flow, after subscribing to the same uplink topic of the TTN broker, is in charge of:

- Retrieving through the internet the data received and published by the TTN broker exploiting the light blue TTN Uplink Node producing an output Node.js buffer;
- Converting this Node.js buffer to a string;
- Parsing this string by exploiting two function nodes featuring JavaScript codes, dedicated to Water Content and Temperature, respectively, which also compose the query for the database;
- Sending the query to the MySQL database running on the Virtual Machine through a dedicated TCP port (internet connection through MySQL 3306 port) employing the orange node.

Moreover, in the second Node-RED sub-flow the application is allowed to transmit downlinks to TTN (i.e., to the device) when the bistable solenoid valve must be actuated. This is accomplished by publishing on a specific downlink topic of the TTN broker using the internet again. In the NodeRED flow, the first “TCP in” node is ready to receive messages on a given unassigned TCP port, then a “Reply” JavaScript function returns an object which contains the ID of the target node and the payload, i.e., the message sent by the server. The last light blue node is a TTN Downlink Node which publishes these data on the TTN broker.

Our LoRaWAN™ Gateway is composed of a Raspberry Pi, an iC880a concentrator able to receive packets of different end devices simultaneously sent with different spreading factors on up to 8 different channels in parallel, and an interconnecting backplane (Figure 7b). The embedded software of the Gateway is proprietary and supplied by TTN. The gateway receives LoRa packets from nodes and forwards them to The Things Network [59] through the MQTT protocol thanks to a wideband network, typically WiFi or Ethernet build. On the other hand, it is well known that for data transmission, MQTT could rely on the TCP protocol but a variant, MQTT-SN, is used over other transports such as UDP (or even Bluetooth). However, TTN does not specify which transport protocol is exploited in its Raspberry Pi firmware.

### 3.3. The Virtual Machine in the Cloud, Database Application, and Graphical User Interface

**Figure 8:**
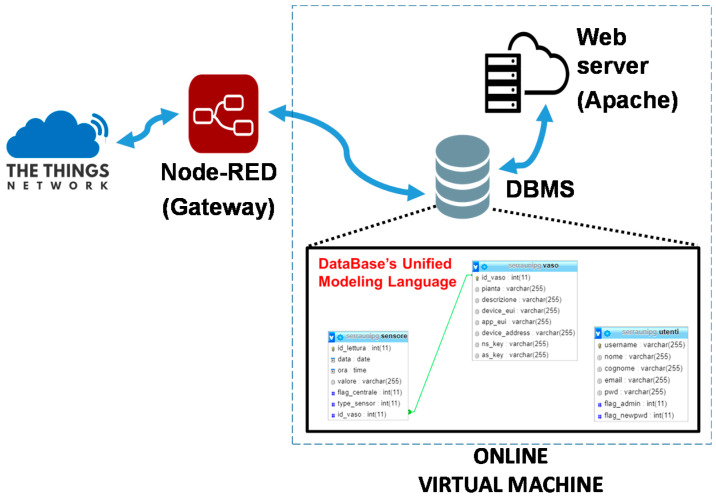
Block diagram of the virtual machine.

At the application level, we installed a Linux virtual machine (Figure 8) that includes:

- Web site (HTML, PHP, CSS, and JavaScript) within a web server;
- MySQL Database Management System (DBMS) server.

The database is divided into two units: (i) node data section and (ii) web application user data section (e.g., username and password). The node data section is further composed of two tables: the first one identifies the node and the second one the sensor with its data. Finally, the webserver fetches data in the database using PHP and shows them on a web page. CanvasJS is used for the Graphical User Interface (GUI). CanvasJS is described as a JavaScript Charting Library for High Performance and ease of use. It is built using the Canvas element and it can render thousands of data points in a matter of milliseconds. CanvasJS is also interactive and can be updated dynamically. Examples of the GUI, operating both from a PC and a smartphone, can be found in the following sections.

The information contained in the database of our virtual machine could represent a starting point for decision-making processes supporting smart monitoring in the frame of PA. A possible implementation could be to develop and enhance the PHP code, used until now to retrieve information from the database, adding a new section where data are analyzed by a dedicated algorithm. The decisions made by the algorithm could be directly sent to the nodes through TTN and the gateways. As an alternative, the watering decision could be directly issued by an application running on the mobile device of the greenhouse manager.

**Figure 9:**
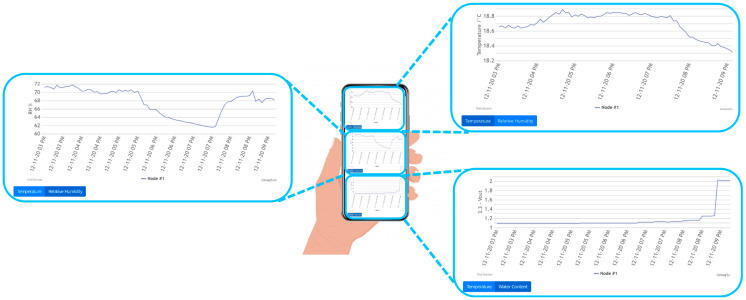
Simultaneous acquisition of ambient temperature and ambient relative humidity (RH) of the greenhouse node and soil water content of a single plant node, shown on the display of a portable device.

A plant node was placed in a pot hosting a daisy plant, while a greenhouse node was acquiring data in the ambient. Figure 9 includes three plots of our JavaScript GUI showing the soil water content recorded by the Plant Node together with the ambient relative humidity and temperature recorded by the greenhouse node in the same room where the plant pot was located.

## 4. Materials

The functionality and reliability of the whole system were proven during two continued experiments with two different natural soils, characterized by very different soil hydraulic properties (see Table 2).

**Figure 10:**
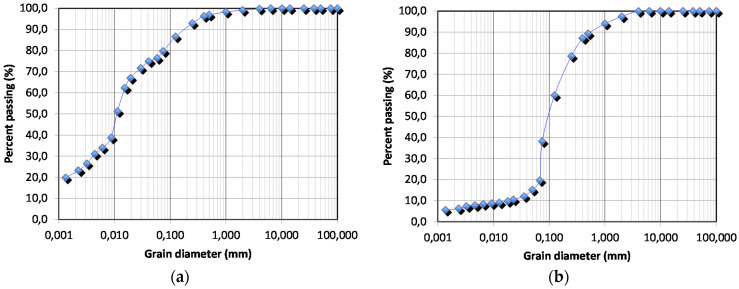
Grain size distribution of the soils used for the experiments: (**a**) fine-textured soil (Silty Loam, according to USDA); (**b**) coarse-textured soil (Loamy Sand, according to USDA).

The fine-textured soil (a Silty Loam, according to the United States Department of Agriculture, USDA, classification [68,69]) was composed of 1% gravel, 22% sand, 54% silt, and 23% clay (Figure 10a), while the coarse-textured soil (a Loamy Sand, according to USDA) was composed of 4% gravel, 79% sand, 11% silt, and 6% clay (Figure 10b).

## 5. Methods, Tests, and Results

Focusing on the Plant Nodes, the system has been tested during a continued experiment where the two different greenhouse soils were watered several times, to verify if the sensor was able to reliably acquire, transmit, and store the ambient temperature and the soil water content parameters in real time and to show them on the custom GUI.

**Figure 11:**
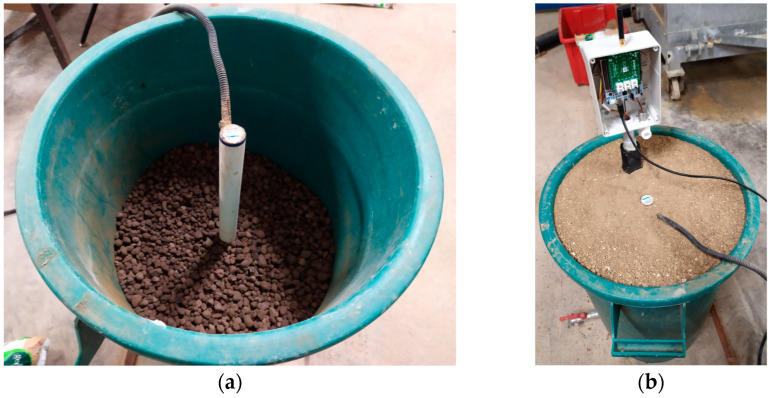
The plastic box used for our experiments. (**a**) Expanded clay aggregate bottom filler, together with a Sentek Drill & Drop Probe. (**b**) Plant Node #1 and Sentek sensor during acquisition.

The measurements were made in a plastic box initially filled with expanded clay aggregate which allowed percolated water to outflow and where a Sentek Drill & Drop Probe (hereafter named “reference sensor”) was driven (Figure 11a). Then the remaining top 30 cm of the box was filled with the chosen soil, either Loamy Sand or Silty Loam. Both soils were packed in 0.05 m lifts and gently tapped into place. This accurate packing mechanism was adopted to achieve homogeneity vertically, to keep perfect contact at the interface, and to minimize preferential flow along the sides of the box. The reference sensor was placed at the center of the box. It features an array of water content and temperature sensors placed at 5 cm, 15 cm, 25 cm, 35 cm, and 45 cm from the top surface.

Then our Plant Node #1 was inserted in the soil at a distance of 10 cm from the Sentek sensor (Figure 11b, where Node #1 is shown without the lid and connected to a 230 ACV-5 DCV adapter during a test measurement). Since the reference sensor and the Plant Node #1 are installed in different positions/depths, this has an impact on the measurements, as explained in the following sections. Data of Plant Node #1 were collected every 5 min for several days while the automatic acquisition system of the reference sensor stored the measurement results every minute. In carrying out the measurements, the soil was watered in consecutive steps.

Before and after each measurement a calibration was performed on the Capacitive Soil Moisture Sensor v1.2 measuring water content, exposing it for 15 min. to air, then dipping it for 15 min in tap water. The reproducibility of these measurements certifies that the low-cost water content is in working order.

### 5.1. Measurements in Silty Loam

**Figure 12:**
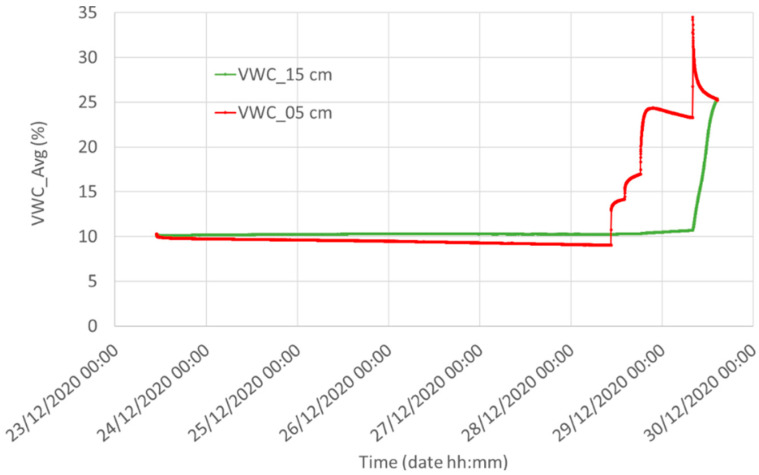
Water content measured by the reference system at two different depths in Silty Loam: red, 5 cm underground, and green, 15 cm underground.

In Figure 12 we compare the water content measured by the reference sensor at a depth of 5 and 15 cm in Silty Loam. After installing the sensors in a uniformly and slightly moistured Silty Loam (initial volumetric water content of 10%), then four synchronous waterings, clearly visible at a depth of 5 cm, were performed during the last two days of this measurement. The two plots witness the strong dependence of the water content on the soil depth in Silty Loam.

**Figure 13:**
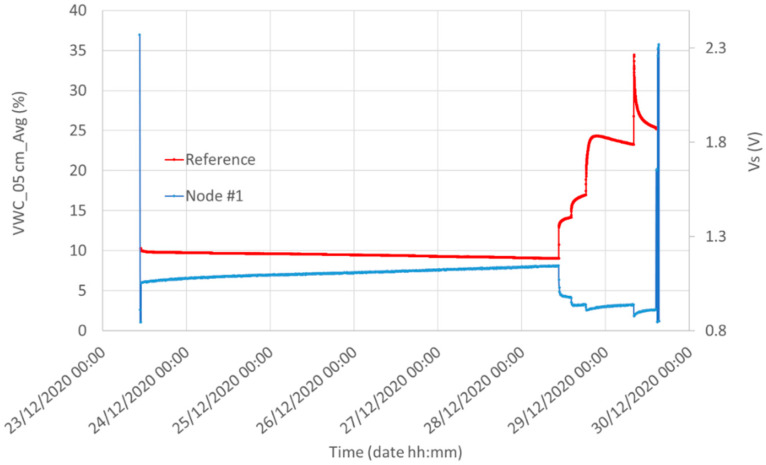
Water content measured by the reference system (red, 5 cm underground) and the Capacitive Soil Moisture Sensor v1.2 (blue) in Silty Loam. Initial and final peaks of the Node #1 plot represent a calibration of the Capacitive Soil Moisture Sensor v1.2 obtained by placing the sensor for 15 min in the air (maximum peak) and 15 min in tap water (minimum peak).

Figure 13 shows the water content measured by the reference system (5 cm underground) and the output voltage of the Capacitive Soil Moisture Sensor v1.2 in Silty Loam (Node #1). The qualitative correlation between the two plots is evident: each watering causes an increase in the measured water content of the reference sensor and a decrease in the output voltage of Node #1. Moreover, the long time elapsed in soil with VWC of about 10% before the first watering guarantees the Capacitive Soil Moisture Sensor v1.2 had plenty of time to reach its settling time. However, we note the lack of linearity of the Capacitive Soil Moisture Sensor v1.2: its sensitivity is too high for small values of water content and it is substantially reduced for volumetric water contents greater than about 15%. The low draining capability of this soil which maintains its water content during the time causes high values of water content and for this reason, the Capacitive Soil Moisture Sensor v1.2 works most of the time almost in saturation. Future improvements for the sensor should be directed towards the linearization of the input/output curve to obtain a constant sensitivity. A detailed discussion of the correlation between the results of the two sensors is reported below.

**Figure 14:**
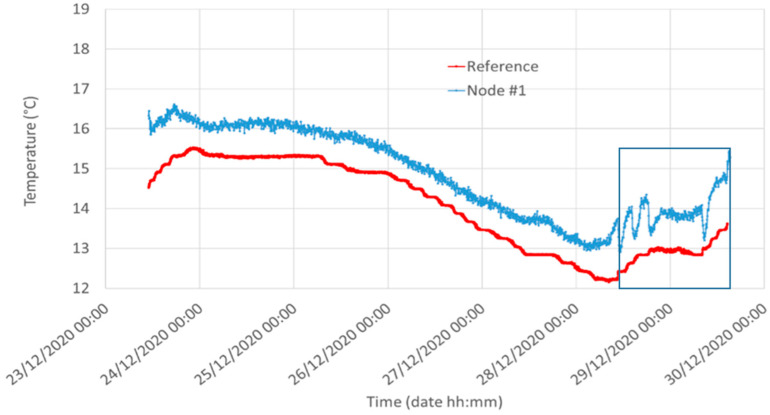
Temperature measured by the reference system (red, 5 cm underground) and the LM35 mounted in Node #1 (blue) in Silty Loam. The rectangular area shows the region where we realized the calibrations of Section 6.2.

Figure 14 shows the reference temperature compared to the temperature of Node #1. In addition, in this case a slight difference is detected, most likely due to the distance of the two sensors and the intrinsic measurement error. Indeed, the LM35 declares a 0.5 °C ensured accuracy (at 25 °C) while the reference Sentek system has a temperature error of 0.1 °C.

### 5.2. Measurements in Loamy Sand

**Figure 15:**
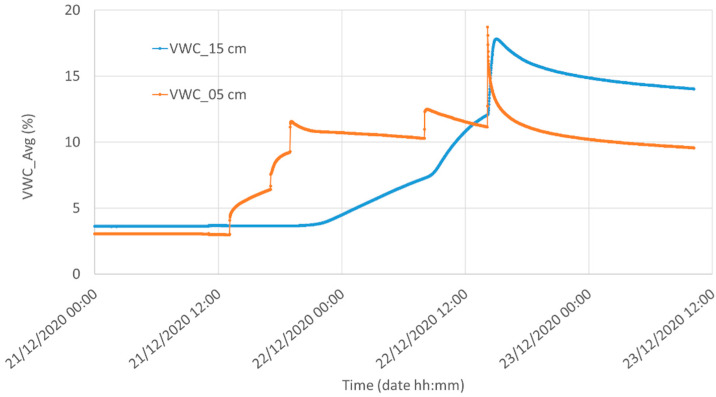
Water content measured by the reference system at two different depths in Loamy Sand: orange, 5 cm underground, and blue, 15 cm underground.

In Figure 15 we compare the water content measured by the reference sensor at a depth of 5 and 15 cm in Loamy Sand. The water content curve at 5 cm clearly shows five consecutive waterings performed during the 2 days of this measurement. On the other hand, the water content curve at 15 cm shows an increase only after the 3rd watering, clearly witnessing the dependence of water content on the soil depth. Furthermore, due to the alternation of rainfall and water redistribution periods, the evolution in time of the wetting front is very complex, as [70,71] showed in their schemes with compound profiles.

**Figure 16:**
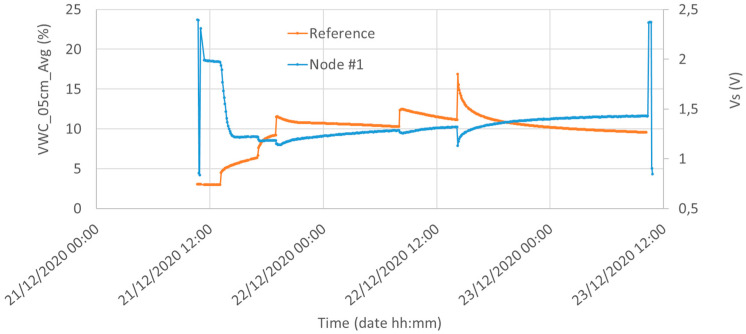
Water content measured by the reference system (orange, 5 cm underground) and the Capacitive Soil Moisture Sensor v1.2 (blue) in Loamy Sand. Initial and final peaks of the Node #1 plot represent a calibration of the Capacitive Soil Moisture Sensor v1.2 obtained by placing the sensor for 15 min in the air (maximum peak) and 15 min in water (minimum peak).

Figure 16 shows the water content measured by the reference system (5 cm underground) and the output voltage of the Capacitive Soil Moisture Sensor v1.2 in Loamy Sand. In addition, for this soil, we obtain a “first sight” reasonable qualitative agreement between the results of the two sensors. Again, we note that the sensitivity of the Capacitive Soil Moisture Sensor v1.2 is too high for small values of water content and it is substantially reduced for volumetric water contents greater than about 10% for this soil material. A detailed discussion of the correlation between the results of the two sensors is reported below.

**Figure 17:**
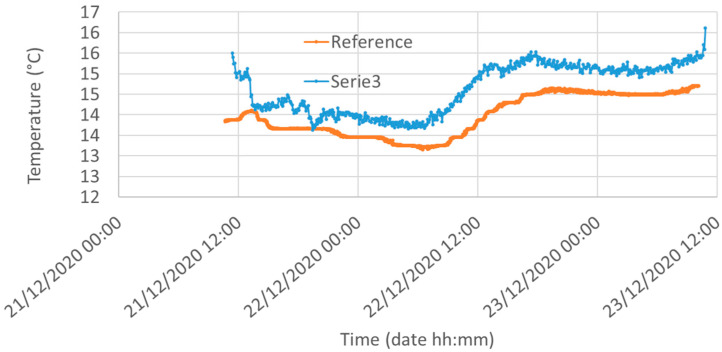
Temperature measured by the reference system (orange, 5 cm underground) and the LM35 mounted in Node #1 (blue) in Loamy Sand.

Figure 17 shows the reference temperature compared to the temperature of Node #1. A slight difference is detected, most likely due to the distance of the two sensors and the intrinsic measurement error.

## 6. Discussion

Measurement outcomes have been discussed in the relevant sections. In this section, we add some comments and considerations that will help to clarify the experimental observations and will allow us to extract the VWC from the output voltage of the Capacitive Soil Moisture Sensor v1.2.

In principle, the results of the reference sensor and the Capacitive Soil Moisture Sensor v1.2 could not exactly be correlated since:

- Node #1 is 10 cm far from the reference sensor and soil compaction and watering could not be perfectly uniform in that area;
- Measurement results from Node #1 could be influenced by temperature variations;
- The Capacitive Soil Moisture Sensor v1.2 measures an average water content of approximately the first 5 cm of the soil where it is inserted, while the reference sensor is placed at 5 cm from the soil surface with a wider thickness of influence (spanning a depth between 0 and 10 cm).

Regarding the first observation, sample preparation described in Section 4 included an accurate packing mechanism to achieve vertical homogeneity. However, this sample preparation does not guarantee uniform compaction of the soil. In [8] we demonstrated that compaction has an obvious strong influence on the results of the Capacitive Soil Moisture Sensor v1.2. The 10 cm distance between the reference sensor and the Capacitive Soil Moisture Sensor v1.2 could affect water content measurement accuracy and cause a discrepancy between the reference and the low-cost sensor. Non-uniform watering is a second source of non-uniformity, even if watering was manually performed trying to evenly distribute water. Therefore, non-uniform soil compaction and watering represent a random added error to our measurements, which should be kept at a minimum using experience and best practices.

Regarding the temperature variations during the measurements, temperature compensation of VWC could be feasible. This task has been demonstrated to be necessary in the case of a temperature spanning about 20 °C [27]. In that case, backpropagation neural networks have been successfully adopted for correcting the soil moisture information from a low-cost sensor using soil temperature data. However, in our experiment, the temperature variations are significantly smaller, about 2 °C, and a correction was not implemented.

Differently from the previous sources of error, the possible error due to different depths of the reference and the low-cost sensor could be taken into account by properly modeling infiltration and redistribution of water during and after rainfall [69,70,71] as explained in the next subsections. In detail, in Section 6.1 we introduce a consolidated infiltration model available in the literature to obtain the soil water content at any depth, whereas in Section 6.2 we correlate the described Capacitive Soil Moisture Sensor v1.2 output voltage with the prediction of the infiltration model for the two different soils.

### 6.1. The Modeling Infiltration and Redistribution of Water

To obtain the soil water content at any depth, *z*, the Corradini et al. [72] infiltration model was used (hereafter named “C et al. (97)”). As shown by Melone et al. [70,71], this model can accurately represent the infiltration process during complex rainfall patterns involving rainfall hiatus periods.

The model was derived considering a constant value of the initial soil water content, *θ_i_*, and combining the depth-integrated forms of the Darcy law and continuity equation [72]. In addition, as the event progresses in time, *t*, a dynamic wetting profile, of the lowest depth *Z* and represented by a distorted rectangle through a shape factor *β*(*θ*_0_) ≤ 1, was assumed. The resulting ordinary differential equation is

(4) $$\frac{d\theta_{0}}{dt}=\frac{\left(\theta_{0}-\theta_{i}\right)\beta \left(\theta_{0}\right)}{F^{\prime }\left[\left(\theta_{0}-\theta_{i}\right)\frac{d\beta \left(\theta_{0}\right)}{d\theta_{0}}+\beta \left(\theta_{0}\right)\right]}\left[q_{0}-K_{0}-\frac{\left(\theta_{0}-\theta_{i}\right)G\left(\theta_{i},\theta_{0}\right)\beta \left(\theta_{0}\right)pK_{0}}{F^{\prime }}\right]$$

where *p* is a parameter linked with the profile shape of the soil water content, *θ*, *θ*_0_ is the soil water content at the surface, *K*_0_ is the hydraulic conductivity at the soil surface, *F*′ is the cumulative dynamic infiltration amount, and *G*(*θ_i_*,*θ*_0_) is expressed by the following equation:

(5) $$G=\frac{1}{K_{s}}\overset{\theta_{s}}{\underset{\theta_{i}}{\int }}D\left(\theta \right)d\theta$$

where *θ*_0_ and *K*_0_ were replaced by *θ_s_* and *K_s_*, with *K_s_* the saturated hydraulic conductivity. D(θ) is the soil water diffusivity, defined by $D\left(\theta \right)=K\left(\theta \right)\frac{\partial \psi }{\partial \theta }$, where *K* is the hydraulic conductivity and *ψ* the soil water matric potential. Equation (4) can be applied until a second rainfall pulse happens, with the profile shape of *θ*(*z*) approximated [72] by

(6) $$\frac{\theta \left(z\right)-\theta_{i}}{\theta_{0}-\theta_{i}}=1-exp\left[\frac{\beta z\left(\theta_{0}-\theta_{i}\right)-F^{\prime }}{\left(\beta -\beta^{2}\right)-F^{\prime }}\right]$$

Functional forms for *β* and *p* were obtained by calibration using results provided by the Richards equation applied to a generic silty loam soil, specifically:

(7) $$\beta \left(\theta_{0}\right)=0.6\frac{\theta_{s}-\theta_{i}}{\theta_{s}-\theta_{r}}+0.4$$

(8) $$\beta \cdot p=0.98-0.87\;exp\left(-\frac{r}{K_{s}}\right)\;\;\;\;\;\frac{d\theta_{0}}{dt}\geq 0$$

(9) $$\beta \cdot p=1.7\;\;\;\frac{d\theta_{0}}{dt}<0$$

Equation (4) can be solved numerically. For *q*_0_ = *r*, with *F*′ = (*r* − *K_i_*)*t*, it gives *θ*_0_(*t*) until time to ponding, *t_p_*, corresponding to *θ*_0_ = *θ_s_* and *dθ*_0_/*dt* = 0, then after *t_p_*, with *θ*_0_ = *θ_s_* and *dθ*_0_/*dt* = 0, it provides the infiltration capacity (*q*_0_ = *f_c_*) and for the period with *r* = 0, with *q*_0_ = 0, it gives *dθ*_0_/*dt* < 0 thus describing the redistribution process.

**Figure 18:**
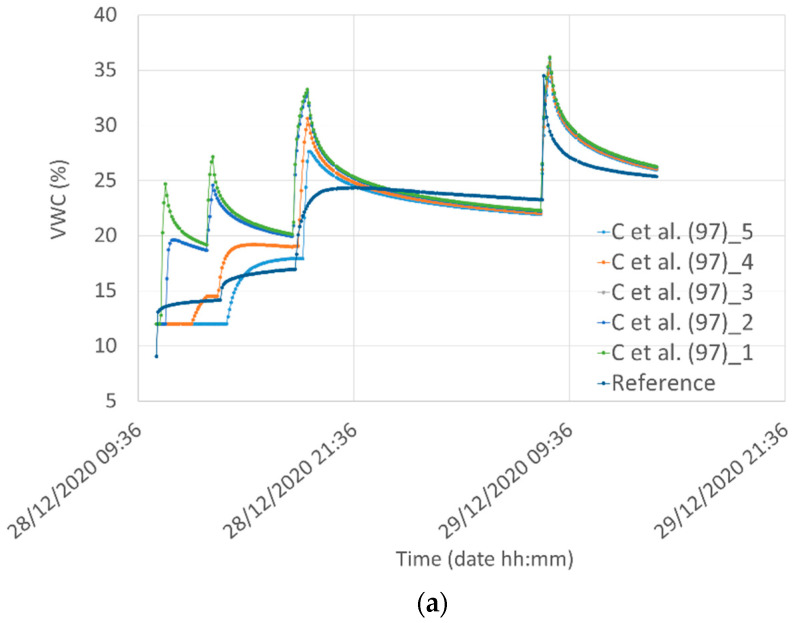

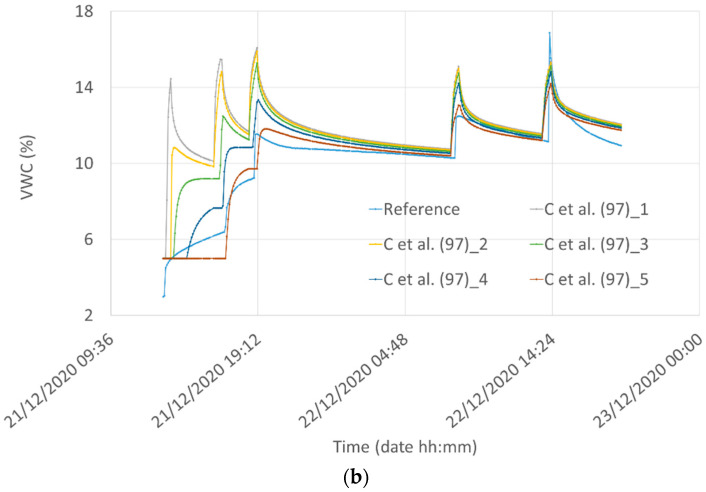
Modeling results for infiltration and redistribution of water (“C et al. (97)” model) during and after rainfall at different depths (1, 2, 3, 4, and 5 cm) for (**a**) Silty Loam and (**b**) Loamy Sand. The model was calibrated with respect to the measured values at a 5 cm depth (Reference sensor). For example, in the figure “C et al. (97)_1” stands for the modeling result at a 1 cm depth.

The involved parameters were estimated through the volume balance criterion along with a best-fit procedure for the water content measured at 5 cm depth by the reference sensor. The initial water contents were set equal to those observed before each experiment, which was found to be almost invariant with depth. Figure 18 shows the results of the model calibration for both the study soils at different depths.

### 6.2. Correlation of the Capacitive Soil Moisture Sensor v1.2 Output Voltage with the Prediction of the Hydraulic Model

In Section 3.1.1 we listed two equations used to correlate and calibrate the output voltage of the Capacitive Soil Moisture Sensor v1.2 with certified water content for two different types of soil: Silty Loam and Loamy Sand. However, the experiments described in the present paper provide a reference water content at an average depth of 5 cm, which is for sure greater than the average detection depth of 2 or 3 cm of the Capacitive Soil Moisture sensor v1.2, which spans a depth from 0 to 5 cm. A possible solution to this problem is to correlate the VWC from Equations (2) and (3) with the extrapolations of the infiltration and distribution Corradini et al. model at a depth of 2 or 3 cm.

In the remainder of the paper, we show the results obtained for the two different soils.

#### 6.2.1. Water Content in Silty Loam

A three-parameter least-square best fit was calculated between the VWC function obtained using the Corradini model (hereafter indicated as “C et al. (97)” [72] at different depths of 2 and 3 cm, with Equation (1), obtaining two triplets of A, B, and C values shown in Table 3 where the Placidi model “P et al. (20)” [8] is referred to different depths. Similarly, a two-parameter least-square best fit was calculated with the Hrisko model “H (20)” at 2 and 3 cm depths and the model from “C et al. (97)”, obtaining two couples of P and Q values shown in Table 3.

**Figure 19:**
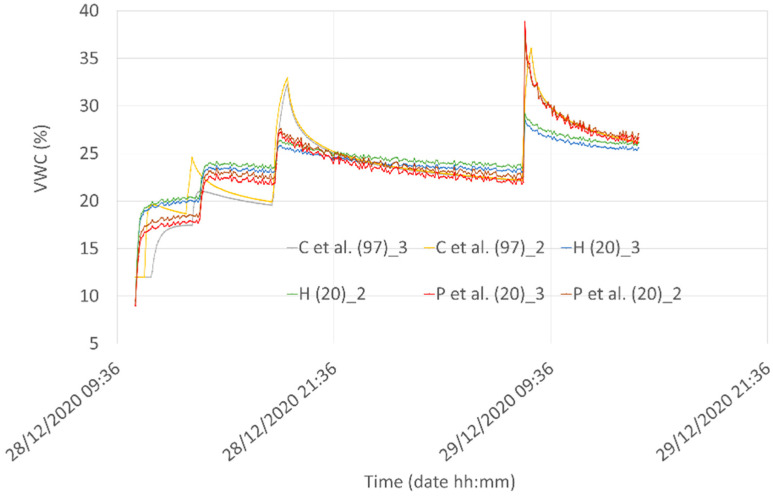
Comparison among the water infiltration and redistribution “C” model for Silty Loam at a depth of 2 and 3 cm with the VWC obtained from the voltage measured by Node #1 using Equation (2) (“P” curves) and Equation (3) (“H” curves).

The plots reporting the water content obtained by using the three models for the two different depths are reported in Figure 19.

**Figure 20:**
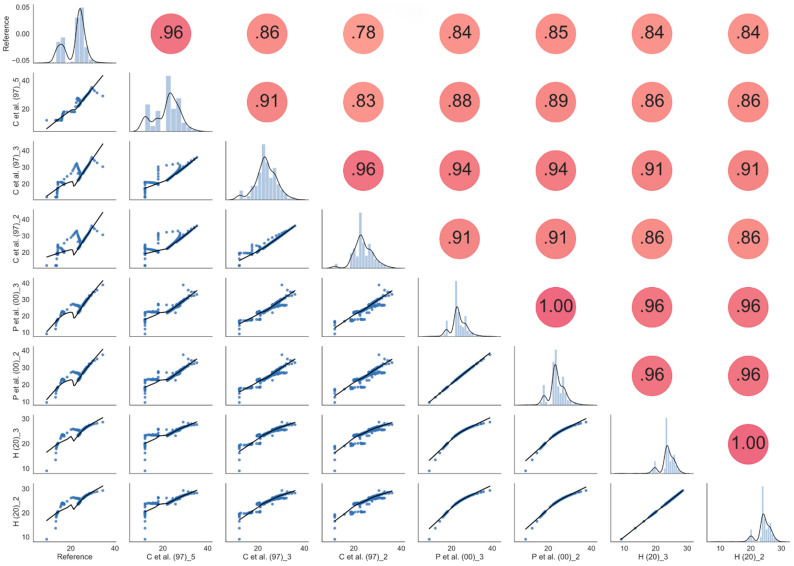
Statistical analysis with scattering plots, cross-correlation values, and kernel density estimation (KDE) obtained by using the Seaborn Python3 tool [73,74,75] in Silty Loam.

In Figure 20 a statistical analysis between all the possible couples of the “C et al. (97)” model and voltage measured by Node #1 using Equation (2) (“P” curves) and Equation (3) (“H” curves) at different depths has been reported. The analysis has been performed by using scattering plots, cross-correlation values, and kernel density estimation accomplished by using the Seaborn Python3 tool [73]. In the figure, the eight plots in the main diagonal are the calculated histograms of the corresponding eight quantities, together with the estimated Gaussian mixture probability density function. The plots in the lower triangular part represent the scattering plots of each couple of quantities, together with the locally weighted regression curve whereas the values in the upper triangular part, instead, represent the correlation coefficients between each couple of quantities.

**Figure 21:**
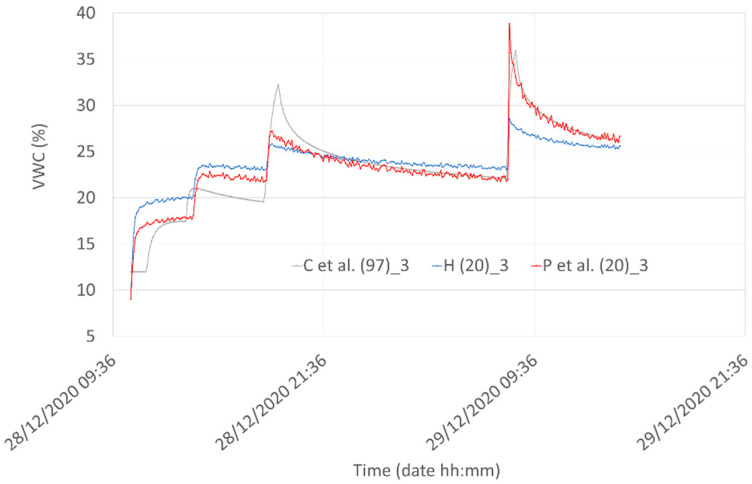
Comparison among the best results obtained from the correlation procedure for the three considered models.

Looking at homogeneous values (i.e., correlation data obtained at the same depth), The best correlation values we obtained (0.94) are between “C et al. (97)” and “P et al. (20”) at a depth of 3 cm. The 0.94 correlation coefficient is slightly greater than the value of 0.91 obtained between “C et al. (97)” and “P et al. (20)” at a depth of 2 cm. In Figure 21 the comparison among the best results obtained from the correlation are reported for the three models. Even if peaks and valleys of the hydraulic model are not always perfectly reproduced by the Capacitive Soil Moisture Sensor v1.2 fitting equations, the overall behavior of the “P et al. (20)” model can capture the main features of the VWC at a shallow depth. We note that, due to the peculiarities of experimental systems involving natural soils, it is impossible to obtain results that are completely reproducible from mathematical schemes. For example, inserting different sensors into the soil produces different preferential waterways that can turn out in minimally different results, especially when the experimental behavior is compared with mathematical model performances.

#### 6.2.2. Water Content in Loamy Sand

A three-parameter least-square best fit was also calculated in Loamy Sand between Equation (1) and the VWC function obtained using the “C et al. (97)” model at different depths of 2 and 3 cm. The two triplets of A, B, and C values are reported in Table 4 where the model “P et al. (20)” is referred to different depths. Similarly, a two-parameter least-square best fit was calculated with the Hrisko model “H (20)” at 2 and 3 cm depths and the model from “C et al. (97)”, obtaining two couples of P and Q values shown in Table 4.

**Figure 22:**
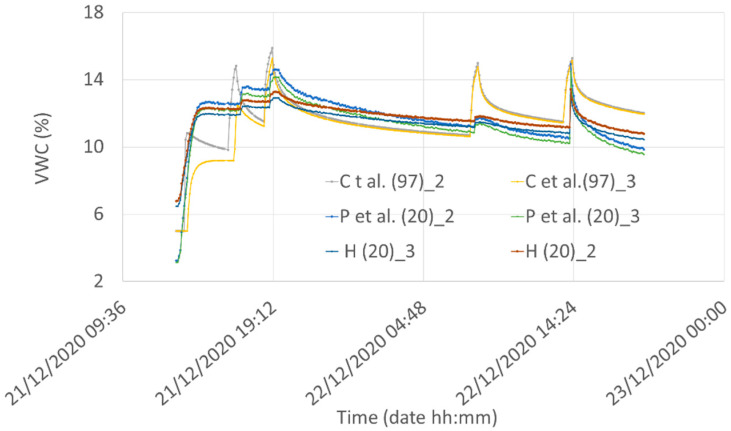
Comparison among the water infiltration and redistribution “C” model for Loamy Sand at a depth of 2 and 3 cm with the VWC obtained from the voltage measured by Node #1 using Equation (2) (“P” curves) and Equation (3) (“H” curves).

The plot with the water content for the three models for the two different depths is reported in Figure 22.

**Figure 23:**
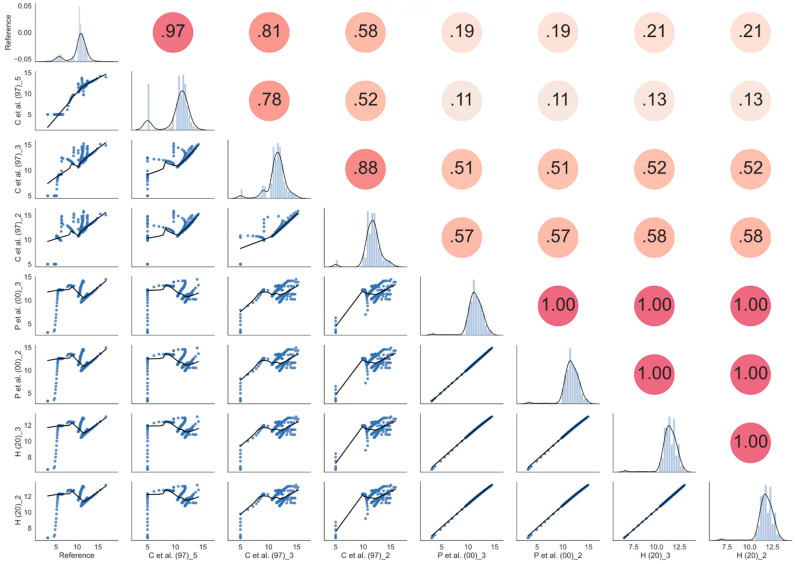
Statistical analysis with scattering plots, cross-correlation values, and kernel density estimation (KDE) in Loamy Sand.

Then a statistical analysis with scattering plots, cross-correlation values, and kernel density estimation was accomplished by using the Seaborn Python3 tool (Figure 23) between all the possible couples of models at different depths. Looking at homogeneous values (i.e., correlation data obtained at the same depth), the best correlation values we obtained (0.58) are between “C et al. (97)” and the “Hrisko model”. A much worse correlation was obtained for Loamy Sand compared to Silty Loam. However, as highlighted in [54], the behavior of coarse-textured soil (as the Loamy Sand) can be mathematically modeled with greater difficulty than that of fine-textured soil (as the Silty Loam).

**Figure 24:**
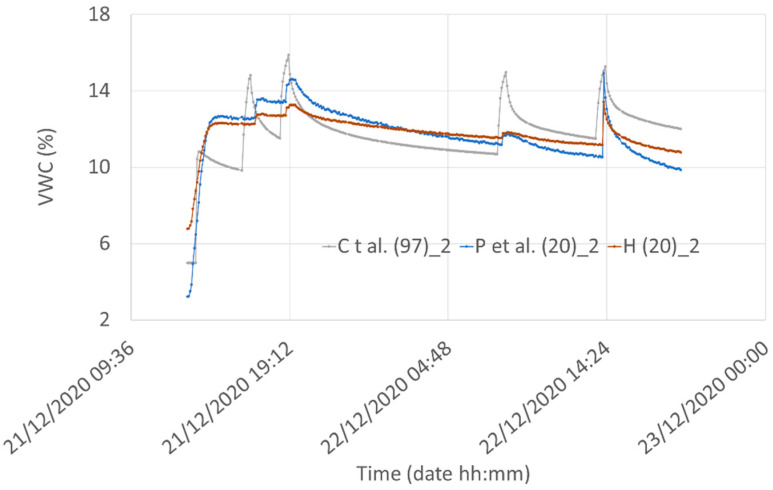
Comparison among the best results obtained from the correlation procedure for the three considered models.

Figure 24 shows the comparison among the best results obtained from the correlation procedure. Even if a first sight comparison of the three curves shows significant differences, it should be noted that for practical applications of sensors for measuring the soil water content, differences of a few percent are often irrelevant.

## 7. Conclusions

A low-power WSN based on LoRaWAN™ was designed with a focus on low-cost PA applications, such as greenhouse sensing and actuation. Two types of wireless nodes were envisaged, greenhouse node and plant node, and the whole LPWAN was designed and implemented, including an 8-channel gateway/concentrator. The first experimental results were collected and stored in a database managed by a virtual machine running in a cloud service. Since all subsystems adopted in this research are off-the-shelf elements with available open-source software libraries, only a minimal effort is needed when the system is implemented for a different application.

Measurement results were focused on measurements of water content and were collected using plant nodes in Loamy Sand and Silty Loam, proving the functionality and reliability of the whole system (sensor nodes, gateway, GUI, Node-RED, and Cloud) and comparing the system behavior with a reference sensor from Sentek. Temperature measurements of our plant nodes compare as expected with the reference sensor within the supposed accuracy of the adopted sensors. Regarding water content measurements, a correlation was attempted between the results of the cheap Capacitive Soil Moisture Sensor v1.2 and those of the Sentek reference sensor. We realized the low-cost water content sensor suffers from a non-constant sensitivity; therefore, non-linear fitting equations are necessary for correlating its voltage output with the VWC. We adopted for the Capacitive Soil Moisture Sensor v1.2 two VWC fitting equations taken from the literature. Since the reference sensor and the cheap water content sensor span different soil depths (5 cm and 2-3 cm, respectively), we first modeled the theoretical VWC profiles at different depths using a proven water infiltration and redistribution model, calibrating the model on the reference sensor results at a depth of 5 cm. Then we used the two fitting equations for the Capacitive Soil Moisture Sensor v1.2 and calculated multi-parameter least squares fit to the hydraulic model at 2 and 3 cm depths. A very satisfactory correlation coefficient of 0.94 was obtained for Silty Loam using the exponential/logarithmic “P” model at a depth of 3 cm. Instead, the best correlation value we obtained using the same fitting procedure applied to the results in Loamy Sand was 0.58 at a depth of 2 cm using the hyperbolic “H” model. Despite the low correlation coefficient, the VWC values we obtained with the hyperbolic “H” model can be considered as representative of the real VWC at a depth of 2 cm, since differences of a few percent are often irrelevant for practical applications of sensors for measuring the soil water content.

In this work, we demonstrated the lack of linearity of the adopted soil water content sensor. Notwithstanding this lack of linearity, the introduction of the infiltration model and of a dedicated statistical analysis allowed us to extract reliable values of the volumetric water content for both Silty Loam and Loamy Sand. This procedure represents the novelty and the potential of the proposed approach. Therefore, future work will address the optimization of the sensor performance. To this purpose, it will be useful to better understand the behavior of the sensor from simulations and to optimize the layout of the sensor without impacting significantly on the cost, also considering the mechanical integration constraints needed for industrialization.

## Figures and Tables

**Table 1:** Selected sensors characteristics.

Sample ID	DC/%	*f*/MHz
S1	37.12	1.221
S2	35.58	1.533
S5	34.36	1.533
S6	32.93	1.524
S7	34.78	1.552
S9	34.36	1.533
S10	35.00	1.510
S13	35.12	1.535
S14	34.02	1.527

**Table 2:** Main hydraulic properties of study soils. *K_s_* = saturated hydraulic conductivity; *θ_s_* and *θ_r_* = saturated and residual water content, respectively; *b_d_* = bulk density.

	Fine-Textured Soil (Silty Loam)	Coarse-Textured Soil (Loamy Sand)
*K_s_* (mmh^−1^)	10.0	30.0
*θ_s_*	0.420	0.295
*θ_r_*	0.057	0.035
*b_d_* (gcm^−3^)	2.628	2.669

**Table 3:** Least-square best-fit parameters of Equations (2) and (3) in Silty Loam.

	P et al. (20)_3	P et al. (20)_2
*A*	0.711	0.731
*B*	9.72	10.2
*C*	0.864	0.859
	H (20)_3	H (20)_2
*P*	73.4	75.8
*Q*	55.1	57.2

**Table 4:** Least-square best-fit parameters of Equation (2) and Equation (3) in Loamy Sand.

	P et al. (20)_3	P et al. (20)_2
*A*	1.64	1.65
*B*	8.16	8.41
*C*	0.85	0.85
	H (20)_3	H (20)_2
*P*	17.44	17.54
*Q*	2.37	2.1
